# Supplementary material for: Risk Factors for Postoperative Deep Infection after Instrumented Spinal Fusion Surgeries for Degenerative Spinal Disease: A Nationwide Cohort Study of 194,036 Patients
Source: J Clin Med. 2022 Jan 31;11(3):778. doi: 10.3390/jcm11030778 (PMC8836461; doi:10.3390/jcm11030778)
Supplement: Supplementary file 1 [file jcm-11-00778-s001.zip › jcm-1482318-supplementary materials.pdf]

Supplementary Table S1. HIRA general name codes for used prophylactic intravenous antibiotics

Category of antibiotics	Type of antibiotics	ATC code	HIRA general name code
1st gen. cephalosporin	cefazolin sodium 1g	J01DB04	125701BIJ
	ceftezole sodium 1g	J01DB12	128501BIJ
	ceftezole sodium 2g	J01DB12	128502BIJ
	ceftezole sodium 0.5g	J01DB12	128503BIJ
	cefazedone sodium 1g	J01DB06	130001BIJ
	cefazedone sodium 2g	J01DB06	130002BIJ
	cefazedone sodium 0.5g	J01DB06	130003BIJ
	cefradine 1g	J01DB09	130102BIJ
2nd gen. cephalosporin	cefamandole nafate 1g	J01DC03	125401BIJ
	cefmetazole sodium 1g	J01DC09	126501BIJ
	cefmetazole sodium 0.5g	J01DC09	126502BIJ
	cefmetazole sodium 2g	J01DC09	126503BIJ
	cefmetazole sodium 1g	J01DC09	468100BIJ
	cefbuperazone sodium 1g	J01DC13	125801BIJ
	cefbuperazone sodium 0.5g	J01DC13	125802BIJ
	cefminox sodium 1g	J01DC12	126601BIJ
	cefotetan disodium (as cefotetan 1g)	J01DC05	127201BIJ
	cefotetan disodium 1g	J01DC05	482900BIJ
	cefotiam hydrochloride 1g	J01DC07	127301BIJ
	cefotiam hydrochloride 0.5g	J01DC07	127302BIJ
	cefotiam hydrochloride 0.3mg	J01DC07	127303BIJ
	cefotiam hydrochloride 1g	J01DC07	465400BIJ
	cefuroxime sodium 1.5g	J01DC02	129001BIJ
	cefuroxime sodium 0.75g	J01DC02	129003BIJ
	flomoxef sodium 0.5g	J01DC14	159501BIJ
3rd gen. cephalosporin	ceftazidime hydrate 1g	J01DD02	128301BIJ
	ceftazidime hydrate 0.5g	J01DD02	128302BIJ
	ceftazidime hydrate 2g	J01DD02	128303BIJ
	cefodizime sodium 1g	J01DD09	126701BIJ
	cefodizime sodium 0.5g	J01DD09	126702BIJ
	cefotaxime sodium 1g	J01DD01	127101BIJ
	cefotaxime sodium 2g	J01DD01	127102BIJ
	cefotaxime sodium 0.5g	J01DD01	127103BIJ
	cefoxitin sodium 1g	J01DC01	127501BIJ
	cefpiramide sodium 1g	J01DD11	127701BIJ
	cefpiramide sodium 0.5g	J01DD11	127702BIJ
	cefpirome sulfate 1g	J01DE02	127801BIJ
	ceftizoxime sodium 1g	J01DD07	128701BIJ
	ceftriaxone sodium hydrate 1g	J01DD04	128801BIJ
	ceftriaxone sodium hydrate 0.25g	J01DD04	128802BIJ
	ceftriaxone sodium hydrate 2g	J01DD04	128803BIJ
	ceftriaxone sodium hydrate 0.5g	J01DD04	128804BIJ
	ceftriaxone sodium hydrate 1.5g	J01DD04	128805BIJ
	ceftriaxone sodium 1g	J01DD04	468200BIJ

	ceftriaxone sodium 2g	J01DD04	478500BIJ
	cefoperazone sodium 0.5g	J01DD12	329900BIJ
	cefoperazone sodium 1g	J01DD12	557400BIJ
	cefoperazone sodium 0.5g	J01DD12	463100BIJ
4th gen. cephalosporin	cefepime hydrochloride hydrate 1g	J01DE01	126101BIJ
	cefepime hydrochloride hydrate 0.5g	J01DE01	126103BIJ
	cefepime hydrochloride hydrate·L-arginine (as cefepime hydrochloride 1g)	J01DE01	800001BIJ
Other beta-lactams	benzathine penicillin G 120M unit	J01CE08	115502BIJ
	penicillin G potassium crystal 5MI.U	J01CE01	210001BIJ
	piperacillin sodium 2g tazobactam 0.25g	J01CR05	329500BIJ
	piperacillin sodium 4g tazobactam 0.5g	J01CR05	329600BIJ
	amoxicillin sodium 0.5g clavulanate potassium 0.1g	J01CR02	328900BIJ
	amoxicillin sodium 1g clavulanate potassium 0.2g	J01CR02	329000BIJ
	amoxicillin sodium (as amoxicillin 1g)	J01CA04	108201BIJ
	amoxicillin sodium (as amoxicillin 0.5g)	J01CA04	108203BIJ
	ampicillin sodium 0.5g	J01CA01	108603BIJ
	nafcillin sodium 1g	J01CF06	360801BIJ
	amoxicillin sodium 1g sulbactam sodium 0.5g	J01CR02	381300BIJ
	amoxicillin sodium 0.5g sulbactam sodium 0.25g	J01CR02	381500BIJ
	ampicillin sodium 0.5g sulbactam sodium 0.25g	J01CR01	328500BIJ
	ampicillin sodium 1g sulbactam sodium 0.5g	J01CR01	328600BIJ
	piperacillin sodium 1g sulbactam sodium 0.5g	J01CR05	433300BIJ
	piperacillin sodium 2g sulbactam sodium 1g	J01CR05	453200BIJ
	piperacillin sodium 3g sulbactam sodium 1.5g	J01CR05	657600BIJ
Glycopeptide and linezolid	teicoplanin 0.2g	J01XA02	234901BIJ
	teicoplanin 0.4g	J01XA02	234902BIJ
	teicoplanin 0.2g	J01XA02	501000BIJ
	vancomycin hydrochloride 1g	J01XA01	247203BIJ
	vancomycin hydrochloride 0.25g	J01XA01	247204BIJ
	vancomycin hydrochloride 0.5g	J01XA01	247205BIJ
	linezolid 0.6g(2mg/mL)	J01XX08	412930BIJ
	vancomycin hydrochloride 1g	J01XA01	479800BIJ
Lincosamide	lincomycin hydrochloride 0.3g(0.3g/mL)	J01FF02	184230BIJ
	lincomycin hydrochloride 0.6g(0.3g/mL)	J01FF02	184231BIJ
	clindamycin phosphate 0.3g(0.15g/mL)	J01FF01	135630BIJ
	clindamycin phosphate 0.6g(0.15g/mL)	J01FF01	135631BIJ
Macrolide	amikacin sulfate 0.25g(0.125g/mL)	J01GB06	106831BIJ
	amikacin sulfate 0.5g(0.25g/mL)	J01GB06	106833BIJ
	amikacin sulfate 0.5g(5mg/mL)	J01GB06	106834BIJ
	tobramycin 80mg(40mg/mL)	J01GB01	240841BIJ
	tobramycin 0.1g(50mg/mL)	J01GB01	240843BIJ
	azithromycin hydrate (as azithromycin 0.5g)	J01FA10	112734BIJ
	gentamicin sulfate 80mg(40mg/mL)	J01GB03	165136BIJ
	isepamicin sulfate 0.2g(0.1g/mL)	J01GB11	177730BIJ
	netilmicin sulfate 0.15g(0.1g/mL)	J01GB07	200732BIJ

	netilmicin sulfate 50mg(25mg/mL)	J01GB07	200730BIJ
	netilmicin sulfate 0.1g(50mg/mL)	J01GB07	200731BIJ
	netilmicin sulfate 0.15g(75mg/mL)	J01GB07	200733BIJ
	ribostamycin sulfate 0.5g(0.3333g/mL)	J01GB10	223830BIJ
	ribostamycin sulfate 1g(0.3333g/mL)	J01GB10	223831BIJ
	ribostamycin sulfate 1g	J01GB10	223801BIJ
	ribostamycin sulfate 0.5g	J01GB10	223802BIJ
	spectinomycin hydrochloride 2g	J01XX04	230801BIJ
	arbekacin sulfate 75mg(50mg/mL)	J01GB12	360930BIJ
	arbekacin sulfate 0.1g(50mg/mL)	J01GB12	360931BIJ
Carbapenem	meropenem 0.5g	J01DH02	190702BIJ
	meropenem 1g	J01DH02	190703BIJ
	meropenem 2g	J01DH02	190704BIJ
	imipenem hydrate (as imipenem 0.25g)	J01DH51	329300BIJ
	imipenem hydrate (as imipenem 0.5g)	J01DH51	329400BIJ
	imipenem monohydrate 0.5g	J01DH51	466100BIJ
	ertapenem sodium (as ertapenem 1g)	J01DH03	447701BIJ
	doripenem monohydrate (as doripenem 0.25g)	J01DH04	593201BIJ
Quinolone	ciprofloxacin 0.1g(2mg/mL)	J01MA02	134133BIJ
	ciprofloxacin 0.2g(2mg/mL)	J01MA02	134134BIJ
	ciprofloxacin 0.4g(2mg/mL)	J01MA02	134135BIJ
	levofloxacin 0.25g(5mg/mL)	J01MA12	183233BIJ
	levofloxacin 0.5g(5mg/mL)	J01MA12	183234BIJ
	levofloxacin 0.75g(5mg/mL)	J01MA12	183235BIJ
	levofloxacin 1g(5mg/mL)	J01MA12	183236BIJ
	ofloxacin 0.2g(2mg/mL)	J01MA01	203940BIJ
	moxifloxacin hydrochloride (as moxifloxacin 0.4g(1.6mg/mL))	J01MA14	380335BIJ
	gemifloxacin mesylate (as gemifloxacin 0.2g)	J01MA15	442902BIJ
	moxifloxacin 0.4g(1.6mg/mL)	J01MA14	801601BIJ
ETC	aztreonam 1g	J01DF01	113001BIJ
	aztreonam 0.5g	J01DF01	113002BIJ
	colistin sodium methanesulfonate 0.15g	J01XB01	484201BIJ
	colistin sodium methanesulfonate 0.16g	J01XB01	484203BIJ
	sulfamethoxazole 0.4g(80mg/mL) trimethoprim 80mg(16mg/mL)	J01EE01	330000BIJ

Supplementary Table S2. ICD-10 codes for comorbidities including Charlson comorbidities index items and scores

Type of comorbidities	Category	ICD-10 codes	Scores
Included in Charlson comorbidity index	Myocardial infarction	I21, I22, I25.2	1
	Congestive heart failure	I09.9, I11.0, I13.0, I13.2, I25.5, I42.0, I42.5-I42.9, I43, I50, P29.0	1
	Peripheral vascular disease	I70, I71, I73.1, I73.8, I73.9, I77.1, I79.0, I79.2, K55.1, K55.8, K55.9, Z95.8, Z95.9	1
	Cerebrovascular disease	G45, G46, I60-I69, H34.0	1
	Dementia	F00-F03, G30, F05.1, G31.1	1
	Chronic pulmonary disease	I27.8, I27.9, J40-J47, J60-J67, J68.4, J70.1, J70.3	1
	Rheumatologic disease	M05, M06, M31.5, M32-M34, M35.1, M35.3, M36.0	1
	Peptic ulcer	K25-K28	1
	Hemiplegia or paraplegia	G04.1, G11.4, G80.1, G80.2, G81, G82, G83.0, G83.1, G83.2, G83.3, G83.4, G83.9	2
	Diabetes without complication	E10.0, E10.1, E10.6, E10.8	1
	Diabetes with complication	E10.9, E11.0, E11.1, E11.6, E11.8, E11.9, E12.0, E12.1, E12.6, E12.8, E12.9, E13.0 E13.1, E13.6, E13.8, E13.9, E14.0, E14.1, E14.6, E14.8, E14.9	2
	Mild liver disease	B18, K70.0-K70.3, K70.9, K71.3-K71.5, K71.7, K73, K74, K76.0, K76.2-K76.4, K76.8, K76.9, Z94.4	1
	Moderate to severe liver disease	I85.0, I85.9, I86.4, I98.2, K70.4, K71.1, K72.1, K72.9, K76.5-K76.7	3
	Moderate to severe renal disease	I12.0, I13.1, N03.2-N03.7, N05.2-N05.7, N18, N19, N25.0, Z49.0-Z49.2, Z94.0, Z99.2	2
	Any malignancy (including leukemia or lyphoma)	C00-C26, C30-C34, C37-C41, C43, C45-C58, C60-C76 C81-C85, C88, C90-C97	2
	Metastatic solid tumor	C77-C80	6
	Acquired immunodeficiency syndrome	B20-B22, B24	6
Others	Osteoporosis	M80-M82	-
	End stage renal disease	E10.22, E11.22, E12.22, E13.32, E14.22, N18.5, Z99.2 (V001, V003)	-

Supplementary Table S3. HIRA therapeutic codes for transfusion

Type of transfusion	HIRA therapeutic code
Autologous transfusion	X6001-X6008
Allogenous transfusion	X2021, X2022, X2031, X2032, X2091, X2092, X2111, X2131, X2132, X2512, X2515, X9006-9007

Supplementary Table S4. HIRA general name codes for used steroids

Category of steroid	Type of steroid	ATC code	HIRA general name code
Oral steroid	deflazacort 6 mg	H02AB13	140801ATB
	dexamethasone 0.5 mg	H02AB02	141901ATB
	dexamethasone 0.75 mg	H02AB02	141903ATB
	betamethasone 0.25 mg + d-chlorpheniramine 2 mg	H02AB01	296900ATB
	hydrocortisone 10 mg	H02AB09	116401ATB
	hydrocortisone 5 mg	H02AB09	170901ATB
	methylprednisolone 4 mg	H02AB04	193302ATB
	methylprednisolone 1 mg	H02AB04	193305ATB
	prednisolone 5 mg	H02AB06	217001ATB
	triamcinolone 1 mg	H02AB08	243201ATB
	triamcinolone 2 mg	H02AB08	243202ATB
	triamcinolone 4 mg	H02AB08	243203ATB
	fludrocortisone 100 μg	H02AA02	160201ATB
Intravenous steroid	dexamethasone 4 mg	H02AB02	142030BIJ
	dexamethasone 4 mg	H02AB02	142230BIJ
	dexamethasone 5 mg	H02AB02	142232BIJ
	betamethasone 4 mg	H02AB01	116530BIJ
	hydrocortisone 100 mg	H02AB09	171201BIJ
	methylprednisolone 125 mg	H02AB04	193601BIJ
	methylprednisolone 40 mg	H02AB04	193603BIJ
	methylprednisolone 500 mg	H02AB04	193604BIJ
	triamcinolone 10 mg	H02AB08	243336BIJ
	triamcinolone 40 mg	H02AB08	243335BIJ
	triamcinolone 40 mg	H02AB08	243337BIJ

Supplementary Table S5. (1) Risk factors for postoperative infection after instrumented spinal fusion among the patients with over 2-week antibiotics (model 1). (2) Risk factors for postoperative infection after instrumented spinal fusion among the patients with over 2-week antibiotics (model 2).

(1)

Variables	Category	Model 1			
		Adjusted odds ratio (95% confidence interval)	p-value	Bootstrap adjusted odds ratio (95% confidence interval)	Relative bias (%)
Age	50-59 vs 20-49	1.1 (0.8 - 1.5)	0.610	1.1 (0.9 - 1.4)	-5.3
	60-69 vs 20-49	1.3 (1.0 - 1.8)	0.051	1.3 (1.1 - 1.7)	0.9
	70-79 vs 20-49	1.4 (1.1 - 1.9)	0.021	1.4 (1.1 - 1.9)	-0.1
	80+ vs 20-49	1.9 (1.3 - 2.7)	<0.001	1.9 (1.5 - 2.6)	1.1
Sex	Male vs female	1.8 (1.6 - 2.1)	<0.001	1.8 (1.6 - 2.1)	1.2
Regions	Rural vs urban	1.3 (1.1 - 1.5)	0.004	1.3 (1.1 - 1.5)	0.0
Surgical approach	Cervical anterior vs lumbar posterior	0.2 (0.1 - 0.3)	<0.001	0.2 (0.1 - 0.2)	1.5
	Cervical posterior vs lumbar posterior	0.4 (0.2 - 0.7)	0.005	0.3 (0.2 - 0.6)	10.5
	Thoracic anterior vs lumbar posterior	1.4 (0.2 - 10.3)	0.725	0.1 (0.0 - 4.2)	-841.8
	Thoracic posterior vs lumbar posterior	1.4 (1.0 - 1.9)	0.063	1.4 (1.0 - 1.8)	-6.0
	Lumbar anterior vs lumbar posterior	0.7 (0.4 - 1.5)	0.420	0.7 (0.3 - 1.3)	18.3
	Multiple vs lumbar posterior	1.2 (1.1 - 1.4)	0.003	1.2 (1.1 - 1.4)	-2.3
Cage		1.1 (0.9 - 1.2)	0.410	1.2 (0.9 - 1.2)	14.3
Charlson comorbidity index score	3-5 vs 0-2	0.9 (0.8 - 1.2)	0.760	0.9 (0.8 - 1.1)	38.3
	over 6 vs 0-2	1.2 (0.7 - 1.9)	0.479	1.2 (0.8 -1.6)	-13.5
Comorbidities	Congestive heart failure	1.2 (0.9 - 1.6)	0.173	1.2 (1.0 - 1.5)	-2.1
	Cerebrovascular disease	1.5 (1.2 - 1.8)	<0.001	1.5 (1.2 - 1.7)	-1.0
	Peripheral vascular disease	1.2 (1.0 - 1.5)	0.023	1.2 (1.1 - 1.4)	-1.7
	Chronic pulmonary disease	1.2 (1.0 - 1.3)	0.050	1.2 (1.1 - 1.3)	2.2
	Rheumatologic disease	1.6 (1.3 - 2.0)	<0.001	1.6 (1.3 - 2.0)	-3.0
	Dementia	1.1 (1.3 - 2.0)	0.791	1.0 (0.7 - 1.5)	-52.7
	Peptic ulcer	1.0 (0.9 - 1.2)	0.817	1.0 (0.9 - 1.2)	41.9
	Liver disease	1.4 (1.1 - 1.6)	<0.001	1.4 (1.2 - 1.6)	0.3
	Diabetes	1.4 (1.2 - 1.6)	<0.001	1.4 (1.2 - 1.6)	-1.0
	Hemiplegia or paraplegia	1.6 (1.1 - 2.5)	0.022	1.6 (1.1 - 2.3)	-7.6
	Renal disease	0.9 (0.6 - 1.3)	0.496	0.8 (0.6 - 1.2)	14.5
	End stage renal disease	1.6 (0.8 - 3.0)	0.173	1.5 (0.9 - 2.7)	-3.5
	Malignancy, primary	1.1 (0.8 - 1.5)	0.510	1.1 (0.9 - 1.4)	0.8
	Osteoporosis	1.1 (1.0 - 1.3)	0.122	1.1 (1.0 - 1.3)	1.6
Allogenous transfusion		1.6 (1.4 - 1.9)	<0.001	1.6 (1.4- 1.8)	0.6

Systemic steroid	Within 2 weeks vs no use	1.0 (0.9 - 1.2)	0.529	1.0 (0.9 - 1.2)	-11.8
	Over 2 weeks vs no use	1.3 (1.0 - 1.7)	0.032	1.3 (1.0 - 1.7)	2.2

(2)

Variables	Category	Model 2 (backward)			
		Adjusted odds ratio (95% confidence interval)	p-value	Bootstrap adjusted odds ratio (95% confidence interval)	Relative bias (%)
Age	60-69 vs 20-49	1.4 (1.0 - 1.8)	0.038	1.4 (1.1 - 1.8)	1.3
	70-79 vs 20-49	1.5 (1.1 - 2.0)	0.010	1.5 (1.2 - 2.0)	0.3
	80+ vs 20-49	1.9 (1.3 - 2.8)	<0.001	2.0 (1.5 - 2.7)	1.2
Sex	Male vs female	1.8 (1.6 - 2.0)	<0.001	1.8 (1.6 - 2.0)	1.0
Regions	Rural vs urban	1.3 (1.1 - 1.5)	0.003	1.3 (1.1 - 1.5)	0.1
Surgical approach	Cervical anterior vs lumbar posterior	0.2 (0.1 - 0.3)	<0.001	0.2 (0.1 - 0.3)	1.5
	Cervical posterior vs lumbar posterior	0.4 (0.2 - 0.8)	0.005	0.4 (0.2 - 0.6)	11.0
	Thoracic posterior vs lumbar posterior	1.4 (1.0 - 2.0)	0.041	1.4 (1.0 - 1.9)	-5.6
	Multiple vs lumbar posterior	1.3 (1.1 - 1.5)	<0.001	1.3 (1.1 - 1.4)	-1.6
Comorbidities	Cerebrovascular disease	1.5 (1.3 - 1.8)	<0.001	1.5 (1.3 - 1.7)	-1.6
	Peripheral vascular disease	1.2 (1.1 - 1.5)	0.010	1.2 (1.1 - 1.4)	-2.6
	Chronic pulmonary disease	1.2 (1.0 - 1.4)	0.017	1.2 (1.1 - 1.3)	0.5
	Rheumatologic disease	1.7 (1.4 - 2.1)	<0.001	1.7 (1.4 - 2.0)	-3.1
	Liver disease	1.4 (1.2 - 1.6)	<0.001	1.4 (1.2 - 1.6)	-0.5
	Diabetes	1.4 (1.2 - 1.6)	<0.001	1.4 (1.3 - 1.6)	-1.0
	Hemiplegia or paraplegia	1.7 (1.2 - 2.5)	0.008	1.6 (1.2 - 2.2)	-8.1
Allogenous transfusion		1.6 (1.4 - 1.9)	<0.001	1.7 (1.5 - 1.9)	0.7

All significant independent variables ($p<0.05$) from the univariable analysis were included in the model 1. Relative bias was estimated as the difference between the mean bootstrapped regression coefficient estimates and the mean parameter estimates of model 1 divided by the mean parameter estimates of model 1. All significant independent variables ($p<0.05$) from the univariable analysis were initially included and subsequently chosen by backward stepwise selection in model 2. Relative bias was estimated as the difference between the mean bootstrapped regression coefficient estimates and the mean parameter estimates of model 2 divided by the mean parameter estimates of model 2.

Supplementary Table S6. (1) Risk factors for postoperative infection after instrumented spinal fusion among the patients with over 6-week antibiotics (model 1). (2) Risk factors for postoperative infection after instrumented spinal fusion among the patients with over 6-week antibiotics (model 2).

(1)

Variables	Category	Model 1			
		Adjusted odds ratio (95% confidence interval)	p-value	Bootstrap adjusted odds ratio (95% confidence interval)	Relative bias (%)
Age	50-59 vs 20-49	1.0 (0.6 - 1.5)	0.858	0.9 (0.6 - 1.4)	91.5
	60-69 vs 20-49	1.5 (1.0 - 2.2)	0.053	1.4 (1.0 - 2.0)	-9.1
	70-79 vs 20-49	1.5 (1.0 - 2.2)	0.064	1.4 (1.0 - 2.0)	-9.9
	80+ vs 20-49	1.5 (0.9 - 2.5)	0.092	1.5 (0.9 - 2.3)	-6.7
Sex	Male vs female	1.9 (1.6 - 2.3)	<0.001	1.9 (1.7 - 2.2)	0.2
Regions	Rural vs urban	1.4 (1.1 - 1.7)	0.002	1.4 (1.2 - 1.6)	3.0
Surgical approach	Cervical anterior vs lumbar posterior	0.2 (0.1 - 0.3)	<0.001	0.2 (0.1 - 0.2)	2.4
	Cervical posterior vs lumbar posterior	0.4 (0.1 - 0.9)	0.026	0.3 (0.1 - 0.7)	22.2
	Thoracic anterior vs lumbar posterior	2.4 (0.3 - 17.3)	0.387	0.1 (0.0 - 7.0)	-360.3
	Thoracic posterior vs lumbar posterior	1.3 (0.8 - 2.0)	0.289	1.2 (0.8 - 1.9)	-18.6
	Lumbar anterior vs lumbar posterior	0.9 (0.4 - 2.1)	0.893	0.9 (0.4 - 1.7)	120.6
	Multiple vs lumbar posterior	1.3 (1.1 - 1.6)	0.004	1.3 (1.1 - 1.5)	-5.8
Cage		1.1 (0.9 - 1.3)	0.461	1.1 (0.9 - 1.4)	11.7
Charlson comorbidity index score	3-5 vs 0-2	1.0 (0.8 - 1.4)	0.798	1.0 (0.8 - 1.3)	-32.3
	over 6 vs 0-2	1.3 (0.7 - 2.3)	0.428	1.3 (0.8 - 2.1)	2.3
Comorbidities	Congestive heart failure	1.1 (0.8 - 1.6)	0.591	1.1 (0.8 - 1.4)	3.0
	Cerebrovascular disease	1.6 (1.3 - 2.0)	<0.001	1.6 (1.3 - 1.9)	-2.2
	Peripheral vascular disease	1.3 (1.1 - 1.7)	0.011	1.3 (1.1 - 1.6)	3.3
	Chronic pulmonary disease	1.2 (1.0 - 1.5)	0.044	1.2 (1.0 - 1.4)	-5.9
	Rheumatologic disease	1.5 (1.1 - 2.1)	0.005	1.5 (1.2 - 1.9)	-5.2
	Peptic ulcer	1.0 (0.8 - 1.3)	0.891	1.0 (0.9 - 1.2)	8.3
	Liver disease	1.4 (1.1 - 1.7)	0.005	1.4 (1.1 - 1.6)	-3.6
	Diabetes	1.5 (1.2 - 1.8)	<0.001	1.5 (1.2 - 1.7)	0.9
	Hemiplegia or paraplegia	2.2 (1.4 - 3.5)	0.001	2.2 (1.4 - 3.3)	-0.6
	Renal disease	0.9 (0.6 - 1.5)	0.754	0.9 (0.6 - 1.4)	-1.6
	End stage renal disease	1.9 (0.9 - 4.0)	0.099	1.7 (0.8 - 3.7)	-16.7
	Malignancy, primary	1.2 (0.8 - 1.7)	0.302	1.2 (0.9 - 1.5)	-6.8
	Osteoporosis	1.3 (1.1 - 1.6)	0.013	1.3 (1.1 - 1.5)	2.2
Allogenous transfusion		1.5 (1.2 - 1.7)	<0.001	1.4 (1.3 - 1.7)	-0.2
Systemic steroid	Within 2 weeks vs no use	1.1 (0.9 - 1.3)	0.450	1.1 (0.9 - 1.2)	-9.0

	Over 2 weeks vs no use	1.6 (1.2 - 2.2)	0.004	1.6 (1.2 - 2.1)	0.9
Type of hospital	Tertiary vs others	1.0 (0.8 - 1.3)	0.6862	1.0 (0.9 - 1.3)	7.4
	General hospital vs others	1.2 (1.0 - 1.5)	0.0824	1.3 (1.1 - 1.5)	42.4

(2)

Variables	Category	Model 2 (backward)			
		Adjusted odds ratio (95% confidence interval)	p-value	Bootstrap adjusted odds ratio (95% confidence interval)	Relative bias (%)
Age	60-69 vs 20-49	1.5 (1.0 - 2.2)	0.045	1.5 (1.1 - 2.1)	-0.6
	70-79 vs 20-49	1.5 (1.0 - 2.2)	0.048	1.5 (1.1 - 2.1)	6.1
Sex	Male vs female	2.0 (1.7 - 2.4)	<0.001	1.8 (1.6 - 2.1)	-10.2
Regions	Rural vs urban	1.4 (1.2 - 1.7)	<0.001	1.4 (1.2 - 1.7)	2.4
Surgical approach	Cervical anterior vs lumbar posterior	0.2 (0.1 - 0.3)	<0.001	0.2 (0.1 - 0.2)	2.2
	Cervical posterior vs lumbar posterior	0.4 (0.2 - 0.9)	0.028	0.3 (0.1 - 0.7)	22.8
	Multiple vs lumbar posterior	1.3 (1.1 - 1.6)	0.002	1.3 (1.1 - 1.5)	-4.0
Comorbidities	Cerebrovascular disease	1.7 (1.4 - 2.1)	<0.001	1.7 (1.4 - 2.0)	-0.8
	Peripheral vascular disease	1.4 (1.1 - 1.7)	0.003	1.4 (1.1 - 1.6)	5.0
	Chronic pulmonary disease	1.3 (1.1 - 1.5)	0.010	1.3 (1.1 - 1.5)	-1.3
	Rheumatologic disease	1.6 (1.2 - 2.1)	0.002	1.6 (1.3 - 2.0)	0.8
	Liver disease	1.4 (1.2 - 1.8)	<0.001	1.4 (1.2 - 1.7)	-0.9
	Diabetes	1.5 (1.3 - 1.8)	<0.001	1.5 (1.3 - 1.7)	0.4
	Hemiplegia or paraplegia	2.4 (1.5 - 3.7)	<0.001	2.4 (1.5 - 3.4)	0.1
	End stage renal disease	1.9 (1.0 - 3.7)	0.040	1.8 (0.9 - 3.1)	-15.5
Allogenous transfusion		1.5 (1.2 - 1.7)	<0.001	1.5 (1.3 - 1.7)	1.0
Systemic steroid	Over 2 weeks vs no use	1.6 (1.2 - 2.2)	0.003	1.6 (1.2 - 2.1)	1.1

All significant independent variables ($p<0.05$) from the univariable analysis were included in the model 1. Relative bias was estimated as the difference between the mean bootstrapped regression coefficient estimates and the mean parameter estimates of model 1 divided by the mean parameter estimates of model 1.

Supplementary Table S7. (1) Risk factors for postoperative infection after instrumented spinal fusion when postoperative infection only included an early infection within 60 days after the index surgery (model 1). (2) Risk factors for postoperative infection after instrumented spinal fusion when postoperative deep infection was defined as an infection that occurred within 60 days after the index surgery (model 2).

(1)

Variables	Category	Model 1			
		Adjusted odds ratio (95% confidence interval)	p-value	Bootstrap adjusted odds ratio (95% confidence interval)	Relative bias (%)
Age	50-59 vs 20-49	1.6 (0.9 - 2.6)	0.097	1.5 (1.0 - 2.4)	-1.5
	60-69 vs 20-49	1.9 (1.1 - 3.1)	0.013	1.9 (1.2 - 3.1)	-0.8
	70-79 vs 20-49	1.9 (1.2 - 3.2)	0.013	1.9 (1.2 - 3.0)	-0.8
	80+ vs 20-49	2.4 (1.3 - 4.5)	0.004	2.5 (1.3 - 4.4)	0.8
Sex	Male vs female	1.8 (1.4 - 2.2)	<0.001	1.7 (1.5 - 2.0)	-1.3
Regions	Rural vs urban	1.2 (1.0 - 1.6)	0.091	1.2 (1.0 - 1.5)	-9.8
Type of hospital	Tertiary vs others	1.0 (0.8 - 1.3)	0.999	1.0 (0.8 - 1.2)	-6509.3
	General hospital vs others	1.0 (0.8 - 1.3)	0.732	1.0 (0.8 - 1.3)	-13.3
Surgical approach	Cervical anterior vs lumbar posterior	0.2 (0.1 - 0.3)	<0.001	0.2 (0.1 - 0.3)	3.4
	Cervical posterior vs lumbar posterior	0.6 (0.2 - 1.5)	0.253	0.5 (0.1 - 1.1)	35.1
	Thoracic anterior vs lumbar posterior	3.9 (0.5 - 28.2)	0.177	0.1 (0.0 - 13.4)	-295.3
	Thoracic posterior vs lumbar posterior	1.6 (1.0 - 2.7)	0.062	1.6 (1.0 - 2.4)	-6.6
	Lumbar anterior vs lumbar posterior	0.9 (0.3 - 2.5)	0.850	0.7 (0.2 - 1.6)	219.8
	Multiple vs lumbar posterior	1.4 (1.2 - 1.8)	0.001	1.5 (1.2 - 1.7)	0.5
Charlson comorbidity index score	3-5 vs 0-2	1.0 (0.6 - 1.4)	0.814	0.9 (0.7 - 1.3)	33.5
	over 6 vs 0-2	1.1 (0.5 - 2.6)	0.755	1.1 (0.5 - 2.1)	-42.7
Comorbidities	Myocardial infarction	2.0 (0.9 - 4.2)	0.085	1.7 (0.8 - 3.2)	-17.8
	Congestive heart failure	1.2 (0.8 - 1.9)	0.369	1.2 (0.8 - 1.6)	-8.5
	Cerebrovascular disease	1.3 (1.0 - 1.8)	0.065	1.3 (1.0 - 1.6)	-5.5
	Peripheral vascular disease	1.3 (1.0 - 1.8)	0.034	1.3 (1.0 - 1.7)	-3.0
	Chronic pulmonary disease	1.3 (1.0 - 1.6)	0.055	1.3 (1.0 - 1.5)	7.2
	Rheumatologic disease	1.7 (1.2 - 2.4)	0.005	1.6 (1.2 - 2.2)	-4.5
	Liver disease	1.5 (1.2 - 2.0)	0.003	1.5 (1.2 - 1.8)	-4.0
	Diabetes	1.4 (1.1 - 1.8)	0.005	1.4 (1.2 - 1.8)	1.2
	Hemiplegia or paraplegia	1.8 (1.0 - 3.4)	0.098	1.7 (0.9 - 2.9)	-5.9
	Osteoporosis	1.1 (0.9 - 1.5)	0.308	1.1 (0.9 - 1.4)	-0.8
Transfusion	Autologous	1.6 (0.7 - 3.9)	0.296	1.3 (0.6 - 3.0)	-43.3
	Allogenous	1.6 (1.3 - 2.0)	<0.001	1.6 (1.4 - 1.9)	0.4
Systemic steroid	Within 2 weeks vs no use	1.1 (0.9 - 1.4)	0.212	1.1 (1.0 - 1.4)	2.6
	Over 2 weeks vs no use	1.4 (0.9 - 2.0)	0.143	1.3 (1.0 - 1.8)	-7.9

(2)

Variables	Category	Model 2 (backward)			
		Adjusted odds ratio (95% confidence interval)	p-value	Bootstrap adjusted odds ratio (95% confidence interval)	Relative bias (%)
Age	60-69 vs 20-49	2.0 (1.2 - 3.3)	0.007	2.0 (1.3 - 3.2)	-0.1
	70-79 vs 20-49	2.1 (1.3 - 3.5)	0.004	2.1 (1.4 - 3.2)	-0.1
	80+ vs 20-49	2.7 (1.5 - 5.0)	0.001	2.7 (1.6 - 4.7)	1.2
Sex	Male vs female	1.7 (1.4 - 2.1)	<0.001	1.7 (1.5 - 2.0)	-1.6
Surgical approach	Cervical anterior vs lumbar posterior	0.2 (0.1 - 0.3)	<0.001	0.2 (0.1 - 0.3)	3.6
	Thoracic posterior vs lumbar posterior	1.7 (1.0 - 2.8)	0.044	1.6 (1.1 - 2.4)	-6.7
	Multiple vs lumbar posterior	1.5 (1.2 - 1.8)	<0.001	1.5 (1.3 - 1.8)	0.3
Comorbidities	Peripheral vascular disease	1.3 (1.0 - 1.7)	0.034	1.3 (1.0 - 1.6)	-2.2
	Rheumatologic disease	1.7 (1.2 - 2.3)	0.004	1.6 (1.2 - 2.1)	-4.1
	Liver disease	1.5 (1.2 - 1.9)	0.002	1.5 (1.2 - 1.8)	-3.6
	Diabetes	1.3 (1.1 - 1.7)	0.005	1.4 (1.1 - 1.6)	2.3
Allogenous transfusion		1.7 (1.3 - 2.0)	<0.001	1.7 (1.4 - 2.0)	0.0

All significant independent variables ($p<0.05$) from the univariable analysis were included in the model 1. Relative bias was estimated as the difference between the mean bootstrapped regression coefficient estimates and the mean parameter estimates of model 1 divided by the mean parameter estimates of model 1. All significant independent variables ($p<0.05$) from the univariable analysis were initially included and subsequently chosen by backward stepwise selection in model 2. Relative bias was estimated as the difference between the mean bootstrapped regression coefficient estimates and the mean parameter estimates of model 2 divided by the mean parameter estimates of model 2.
